# Special Issue: Shiga Toxin-Producing *Escherichia coli*

**DOI:** 10.3390/microorganisms9010001

**Published:** 2020-12-22

**Authors:** Rodney A. Moxley

**Affiliations:** School of Veterinary Medicine and Biomedical Sciences, University of Nebraska-Lincoln, Lincoln, NE 68583-0905, USA; rmoxley1@unl.edu

Globally, Shiga toxin-producing *Escherichia coli* (STEC) is an important cause of diarrheal disease, most notably hemorrhagic colitis, and post-diarrheal sequela, such as hemolytic-uremic syndrome (HUS) [1]. Cattle are a major reservoir of STEC, with approximately half of the cases in humans attributable to foodborne exposure [2]. Prevention of human illness has mainly been through food safety measures [2]. Despite extensive research, no other generally accepted and effective preventive measures or therapies for STEC infections in human patients are available [3]. Many questions remain about STEC virulence factors, pathogenesis, detection, and other aspects that necessitate a continuation of basic and applied research on a wide front. This Special Issue includes 14 papers (nine articles, two communications, one review, one comment, and one reply) that collectively provide novel information on the epidemiology [4,5,6,7], virulence factors [7,8,9,10], and pathogenesis [11,12,13] of STEC, and the molecular structure or toxicity [14,15,16] and immunodetection [17] of Shiga toxin.

A systematic review of STEC in Brazil found no data for 44% of the Brazilian states, highlighting the need for expansion of epidemiological monitoring to the entire country and alignment of food safety standards with that of international bodies [4,5,6]. Although STEC O111:H8 remains the leading serotype in Brazil, a diversity of other serotypes, some carrying virulence genes and belonging to specific sequence types, were isolated from human patients with bloody diarrhea and HUS, indicating the need for further studies to determine whether they have epidemiological relevance [7].

Several studies addressing virulence factors provided novel information about how they influence carriage in reservoir hosts and disease in human patients. A single base pair A to T transversion, intergenic to the curli divergent operons *csgDEFG* and *csgBAC* in *E. coli* O157:H7 stably conferred biofilm formation, epithelial cell invasion, and persistence in cattle [8]. *E. coli* O45:H2 is a close relative, phylogenetically, to *E. coli* O103:H2, sharing a high degree of homology in virulence factors, such as Stx prophages; however, it is distinct from *E. coli* O45:H16, suggesting that serotype O45:H2 may share virulence characteristics of O103:H2, which is frequently associated with severe illness [9]. *E. coli* O157:H7 secretes EF-Tu and L-asparaginase II, with the latter inhibiting T-lymphocyte proliferation [10]. OmpT contributes to *E. coli* O157:H7 adhesion to human epithelial cells [10].

Other studies, using a variety of methods and approaches, extend our knowledge of the pathogenesis of STEC infections. A comparison of the transcriptomic and phenotypic responses of host cells infected with STEC O113:H21 strains from a HUS patient or bovine feces having similar virulence factor profiles, found that the former induced greater and earlier host cell global gene expression with excessive inflammatory and apoptotic responses, which may explain its enhanced virulence [11]. A study reported on the identification of Stx2e receptors on porcine kidney epithelial cells, providing the first data on their Stx2e-mediated damage and suggesting a possible involvement in edema disease of swine [12]. Another study described the first report of *E. coli* O157:H7 causing attaching–effacing lesions in the uroepithelium and the first evidence of the utility of the gnotobiotic piglet as a model for studies of the pathogenesis of STEC-induced urinary tract infections [13].

Studies utilizing top-down proteomic analysis and a protocol for preparation of outer membrane vesicles (OMVs) provide further new information on Stx, and position scientists for future studies to address the effects of Stx on cells. Top-down proteomic analysis of the B-subunit successfully identified both Stx1a and Stx2a in STEC strains with results consistent with whole genome sequencing, validating the utility of this analytical method to distinguish types and subtypes of Stx [14]. A study structurally and functionally characterized Stx2k, a new subtype of Stx2, providing tools for early detection and control of STEC producing this less well-known toxin [15]. A protocol for the preparation of synthetic outer membrane vesicles (OMVs) with a defined lipid composition resembling the *E. coli* outer membrane and loading with functional Stx2a was described [16]. The OMVs were able to deliver Stx2a to host cells in the absence of other confounders of studies of Stx toxicity found in cell membranes—e.g., lipopolysaccharide. This work makes possible future studies on the degree of virulence associated with individual toxins from EHEC and other bacterial pathogens [16].

A study evaluating three immunological diagnostic assays for STEC (latex agglutination, lateral flow, and capture ELISA) found that all were highly sensitive and specific, indicating that robust tools for the diagnosis of STEC infections are available [17]. Collectively, the papers in this Special Issue on STEC reveal significant progress in the understanding of these pathogens, but they also highlight their complexity with the realization that more work is necessary to enable the development of more specific preventive measures and therapies.
